# CESNET-QUIC22: A large one-month QUIC network traffic dataset from backbone lines

**DOI:** 10.1016/j.dib.2023.108888

**Published:** 2023-01-09

**Authors:** Jan Luxemburk, Karel Hynek, Tomáš Čejka, Andrej Lukačovič, Pavel Šiška

**Affiliations:** a Faculty of Information Technology, CTU in Prague, Thakurova 9, Prague, 160 00, Czech Republic; b CESNET, Zikova 4, Prague, 160 00, Czech Republic

**Keywords:** Network monitoring, Traffic classification, Encrypted traffic, QUIC

## Abstract

The QUIC (Quick UDP Internet Connection) protocol has the potential to replace TLS over TCP, which is the standard choice for reliable and secure Internet communication. Due to its design that makes the inspection of QUIC handshakes challenging and its usage in HTTP/3, there is an increasing demand for research in QUIC traffic analysis. This dataset contains one month of QUIC traffic collected in an ISP backbone network, which connects 500 large institutions and serves around half a million people. The data are delivered as enriched flows that can be useful for various network monitoring tasks. The provided server names and packet-level information allow research in the encrypted traffic classification area. Moreover, included QUIC versions and user agents (smartphone, web browser, and operating system identifiers) provide information for large-scale QUIC deployment studies.

**Specifications Table**

**Table:** 

Subject	Computer Networks and Communications
Specific subject area:	Encrypted traffic classification and analysis
Type of data:	Bidirectional network flows in CSV
How the data were acquired:	The network flows were collected in the CESNET2 network, which is a large internet service provider network with around half a million users. The flows were created using the ipfixprobe high-performance flow exporter.
Data format:	Raw
Description of data collection:	The data were collected on the monitoring probes located at the perimeter of the CESNET2 network. Each monitoring probe transmitted flow data to a single flow collector, where QUIC flow filtering, processing, and anonymization were performed. Client IP addresses were anonymized; server IP addresses were left intact as those are public non-sensitive information.
Data source location:	Network: CESNET2, Czech national research and educational network
	Institution: CESNET association
	City: Prague
	Country: Czech Republic
Data accessibility:	Repository name: Zenodo
	Data identification number: 10.5281/zenodo.7409923
	Direct URL to data: https://zenodo.org/record/7409923

## Value of the Data

• The dataset [1] contains one month of QUIC traffic collected from 100 Gbps backbone lines of a large ISP. This unique ISP-based data source provides realistic characteristics of network traffic originating from various web browsers, operating systems, mobile devices, and desktop machines. Also, due to the organic nature of the captured traffic, the dataset covers rich behaviors (for example, all possible user actions and settings) of web services and mobile applications, which would be impossible to achieve in a lab-generated dataset.
• The provided dataset is useful for research in the computer networks field. The real-world nature of data allows researchers to validate and evaluate network traffic classifiers and prepare their algorithms for various traffic phenomena and connection errors that appear in ISP-like networks. Flows in the dataset include packet metadata sequences, which are the standard data input for various tasks in encrypted traffic analysis.
• The provided dataset can be used for (i) the design and real-world evaluation of QUIC web services classifiers [2], [3], [4], (ii) the design and evaluation of traffic type (e.g., streaming, chat, file transfer) classifiers [5], (iii) the dataset can also serve as real-world benign traffic samples in malicious QUIC traffic identification challenges [6], and (iv) thanks to the QUIC user agent field, which is present in 0.2% (still 342 K samples) of dataset flows, it is possible to use the dataset for the recognition of various client devices, operating systems, or web browsers [7].
• There are no public QUIC datasets of comparable size.^1^ Our dataset spans one month, was obtained from 27 TB worth of real-word traffic, comprises over 153 million flows, and has 102 service labels. A high number of class labels is crucial to make the studied classification problems hard and realistic; related work showed that classifiers evaluated on datasets with few classes do not translate well into real-world deployment [8]. Moreover, the long time span of the dataset allows researching other deployment-related problems, such as the QUIC traffic distribution drift [9] and the degradation of the classifier’s performance over time.
• Previous studies [8], [10], [11] have identified the importance of detecting out-of-distribution (OOD) traffic samples. This problem arises when either new web services (mobile applications, protocols, etc.) appear or known web services change their behavior. The OOD detection gives a traffic classifier the power to detect those new or anomalous samples and label them as “unknown”. Apart from the 102 service classes, our dataset includes three background classes, making it suitable for designing and evaluating novel OOD detection approaches for QUIC traffic.

## 1 Objective

The QUIC protocol is gaining adoption across service providers and consumer software. Its usage as the transport protocol in HTTP/3 suggests its future mass usage. However, despite its apparent importance, no extensive QUIC datasets exist. Therefore, we created the CESNET-QUIC22 dataset containing one month of QUIC network traffic transmitted through the backbone lines of CESNET2, which is the Czech national research and educational network that serves half a million people. The real-world nature of the provided traffic and its extensive size create a unique and comprehensive dataset that enables research of the novel yet crucial QUIC protocol.

## 2 Data Description

The dataset consists of network flows describing encrypted QUIC communications. Flows are extended with packet metadata sequences, packet histograms, and with fields extracted from the QUIC Initial Packet, which is the first packet of the QUIC connection handshake. The extracted handshake fields are the Server Name Indication (SNI) domain, the used version of the QUIC protocol, and the user agent string that is available in a subset of QUIC communications.^2^ The next two sections describe two types of data features—the packet sequences, which provide information about the first 30 packets of a connection, and flow statistics, which describe the entire connection.

### 2.1 Packet sequences

Sequences of packet sizes, directions, and inter-packet times are standard data input for traffic analysis. For the packet sizes, we consider payload size after transport headers (UDP headers for the QUIC case). Packet directions are encoded as ±1, where “+1” means a packet sent from client to server, and “−1” a packet from server to client. Inter-packet times depend on the location of communicating hosts, their distance, and on the network conditions on the path. However, it is still possible to extract relevant information that correlates with user interactions and, for example, with the time required for an API/server/database to process the received data and generate the response to be sent in the next packet. Packet sequences have a maximum length of 30, which is the default setting of the used flow exporter.

We also derive three fields from each packet sequence: its length, time duration, and the number of roundtrips. The roundtrips are counted as the number of changes in the communication direction (from packet directions data); in other words, each client request and server response pair counts as one roundtrip.

### 2.2 Flow statistics

Flows in the dataset also include standard flow statistics, which represent aggregated information about the entire bidirectional flow. The fields are: the number of transmitted bytes and packets in both directions, the duration of the flow, and packet histograms. The packet histograms include binned counts of packet sizes and inter-packet times of the entire flow in both directions.^3^ There are 8 bins with a logarithmic scale; the intervals are 0–15, 16–31, 32–63, 64–127, 128–255, 256–511, 512–1024, >1024 [ms or B]. The units are milliseconds for inter-packet times and bytes for packet sizes. Moreover, each flow has its end reason—either it was idle, reached the active timeout, or ended due to other reasons. This corresponds with the official IANA IPFIX-specified values.^4^ The FLOW_ENDREASON_OTHER field represents the forced end and lack of resources reasons. The end of flow detected reason is not considered because it is not relevant for UDP connections.

### 2.3 Dataset structure

**Fig. 1:**
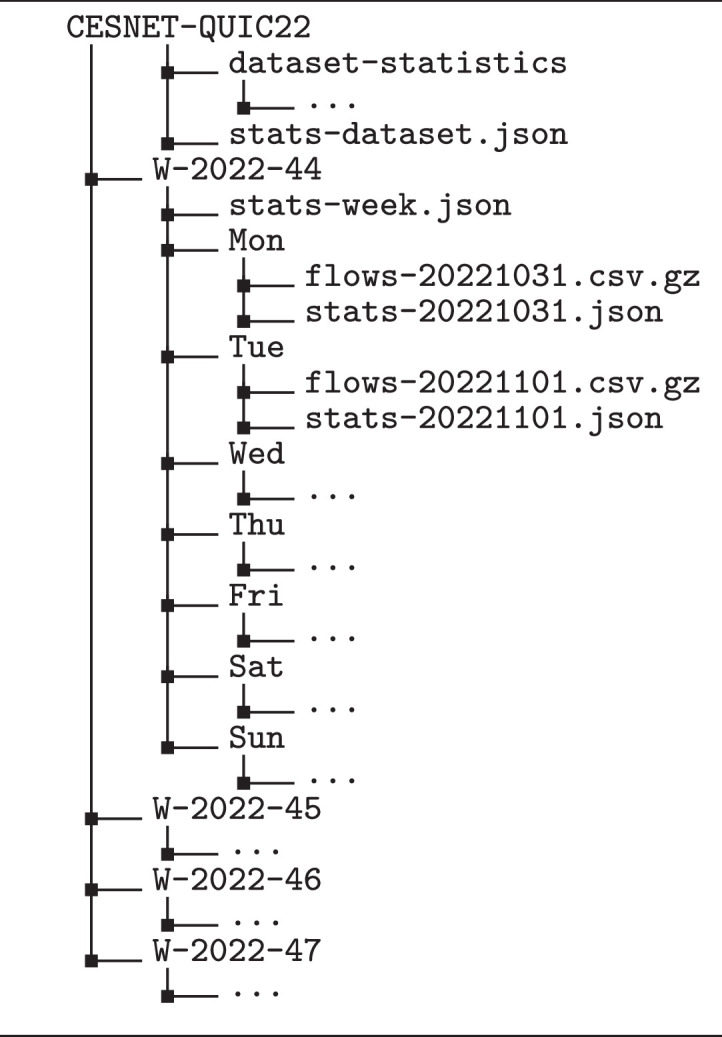
The file structure of the CESNET-QUIC22 dataset.

The dataset flows are delivered in compressed CSV files, which are organized as shown in Fig. 1. CSV files contain one flow per row; data columns are summarized in Table 1. For each flow data file, there is a JSON file with the number of saved and seen (before sampling) flows per service and total counts of all received (observed on the CESNET2 network), service (belonging to one of the dataset’s services), and saved (provided in the dataset) flows. There is also the stats-week.json file aggregating flow counts of a whole week, and the stats-dataset.json file aggregating flow counts for the entire dataset. Flow counts before sampling, which is described in more detail in Section 3.4, can be used to compute sampling ratios of individual services and to resample the dataset back to the original service distribution.

**Table 1:** The description of flow data fields in CSV files.

Column name	Column description
ID	Unique identifier
SRC_IP	Source IP address
DST_IP	Destination IP address
DST_ASN	Destination Autonomous System number
SRC_PORT	Source port
DST_PORT	Destination port
PROTOCOL	Transport protocol^a^
QUIC_VERSION	QUIC protocol version
QUIC_SNI	Server Name Indication domain
QUIC_USER_AGENT	User agent string if available in the QUIC Initial Packet
TIME_FIRST	Timestamp of the first packet in format YYYY-MM-DDTHH-MM-SS.ffffff
TIME_LAST	Timestamp of the last packet in format YYYY-MM-DDTHH-MM-SS.ffffff
DURATION	Duration of the flow in seconds
BYTES	Number of transmitted bytes from client to server
BYTES_REV	Number of transmitted bytes from server to client
PACKETS	Number of packets transmitted from client to server
PACKETS_REV	Number of packets transmitted from server to client
PPI^b^	Packet metadata sequence in the format: [[inter-packet times], [packet diretions], [packet sizes]]
PPI_LEN	Number of packets in the PPI sequence
PPI_DURATION	Duration of the PPI sequence in seconds
PPI_ROUNDTRIPS	Number of roundtrips in the PPI sequence
PHIST_SRC_SIZES	Histogram of packet sizes from client to server
PHIST_DST_SIZES	Histogram of packet sizes from server to client
PHIST_SRC_IPT	Histogram of inter-packet times from client to server
PHIST_DST_IPT	Histogram of inter-packet times from server to client
APP	Web service label
CATEGORY	Service category
FLOW_ENDREASON_IDLE	Flow was terminated because it was idle
FLOW_ENDREASON_ACTIVE	Flow was terminated because it reached the active timeout
FLOW_ENDREASON_OTHER	Flow was terminated for other reasons

a QUIC uses UDP as the transport protocol.

b PPI in field names stands for per-packet information, which is another common name for the packet sequences data.

Moreover, various dataset statistics, such as feature distributions (see Fig. 3) and value counts of QUIC versions and user agents, are provided in the dataset-statistics folder. Table 2 shows per-week flow count, capture period, and uncompressed size.

**Table 2:** Dataset per-week information.

Name	Uncompressed size	Capture period	Flows
W-2022-44	19 GB	31.10.2022–6.11.2022	32.6 M
W-2022-45	25 GB	7.11.2022–13.11.2022	42.6 M
W-2022-46	20 GB	14.11.2022–20.11.2022	33.7 M
W-2022-47	25 GB	21.11.2022–27.11.2022	44.1 M
**CESNET-QUIC22**	89 GB	31.10.2022–27.11.2022	153 M

## 3 Experimental Design, Materials and Methods

The data collection was performed using the monitoring infrastructure of the CESNET association, which is the operator of the CESNET2 national research and educational network. Most of the connected entities are large public organizations such as universities, campuses, high schools, research centers, hospitals, and municipal offices. The CESNET2 network has around half a million users and spans the whole Czech Republic; its topology is shown in Fig. 4.

The monitoring infrastructure of the CESNET2 network follows the traditional IPFIX monitoring approach described in Hofstede et al. [12]. The five monitoring points are located in Prague, Brno, and Ostrava in the Czech Republic. Each is connected to a single or multiple 100 Gbps peering lines via passive optical TAPs. Since the monitoring infrastructure is distributed across multiple machines and locations, system clocks need to be synchronized to ensure accurate time features in monitoring data. The monitoring probes are synchronized using the NTP protocol to a single time server located at CESNET.

The data capture workflow is visualized in Fig. 5. It can be divided into five steps: 1) Service Selection, 2) Flow Enrichment and Export, 3) Flow Collection and Filtration, 4) Flow Sampling, and finally, 5) Anonymization.

### 3.1 Service selection

**Fig. 2:**
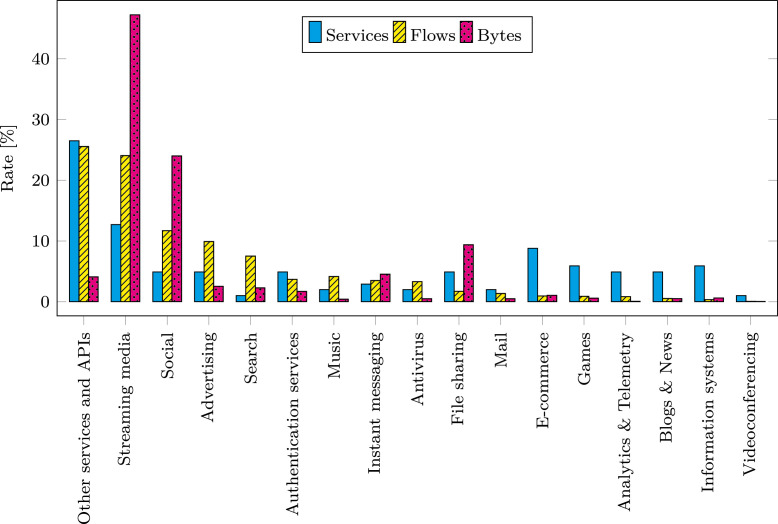
A breakdown of dataset traffic into service categories, showing fractions of services, bytes, and flows.

For building the dataset, we decided to select a diverse and representative subset of web services that could be observed in the CESNET2 network. The selection of the services was based on the following criteria:

**Traffic volume** We prioritized web services with larger traffic volumes so that the dataset covers the majority of real network traffic. The selected web services cover 84% of all QUIC traffic in the CESNET2 network.
**Diversity** We selected diverse web services to capture various types of QUIC traffic. The dataset services can be divided into 17 categories. The distribution of service categories, the number of samples, and the traffic volume per category are shown in Fig. 2.

**Fig. 3:**
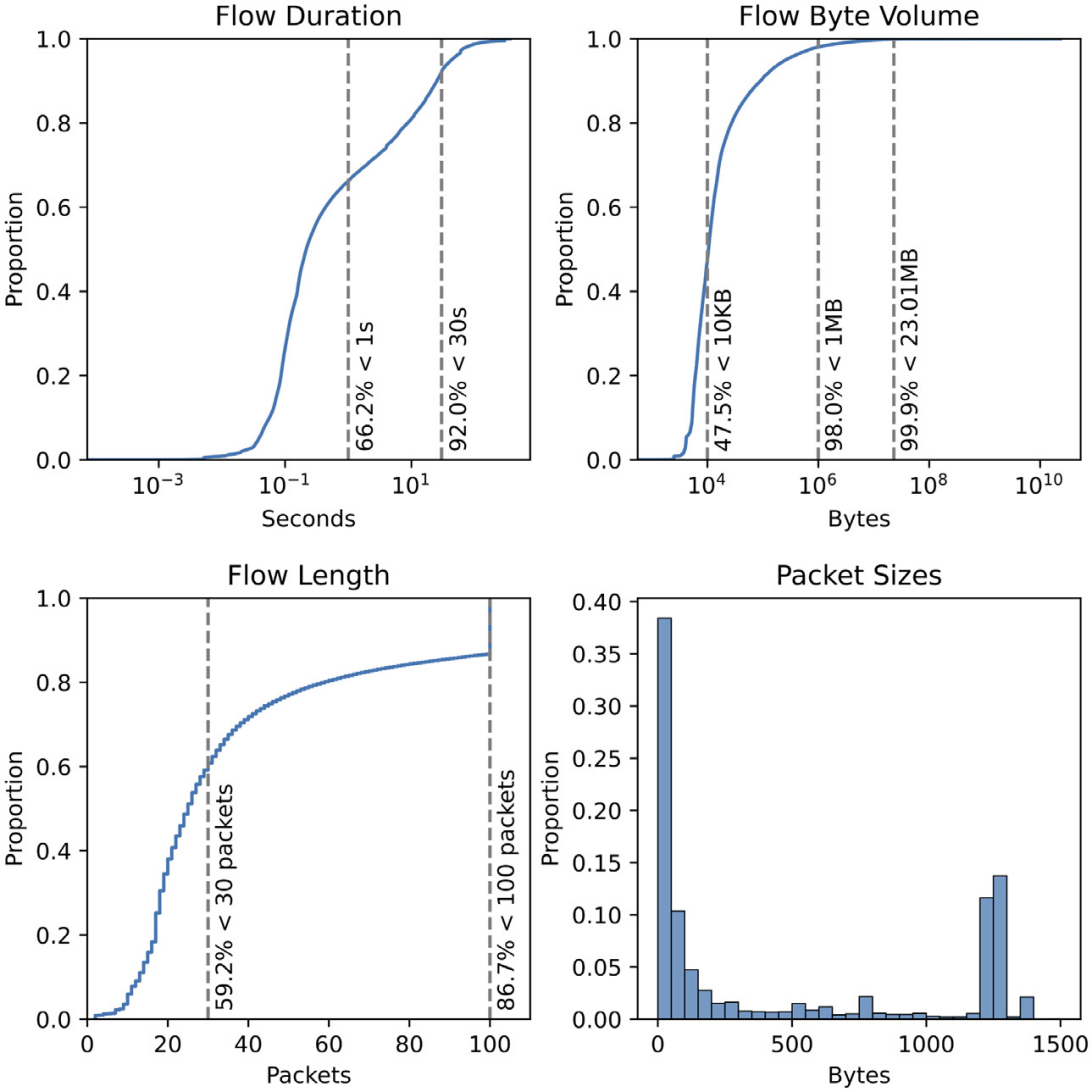
Dataset statistics overview showing cumulative distribution functions of flow duration, byte volume, and packet length. A histogram (50 bin size) of packet sizes across the whole dataset is also shown.

**Fig. 4:**
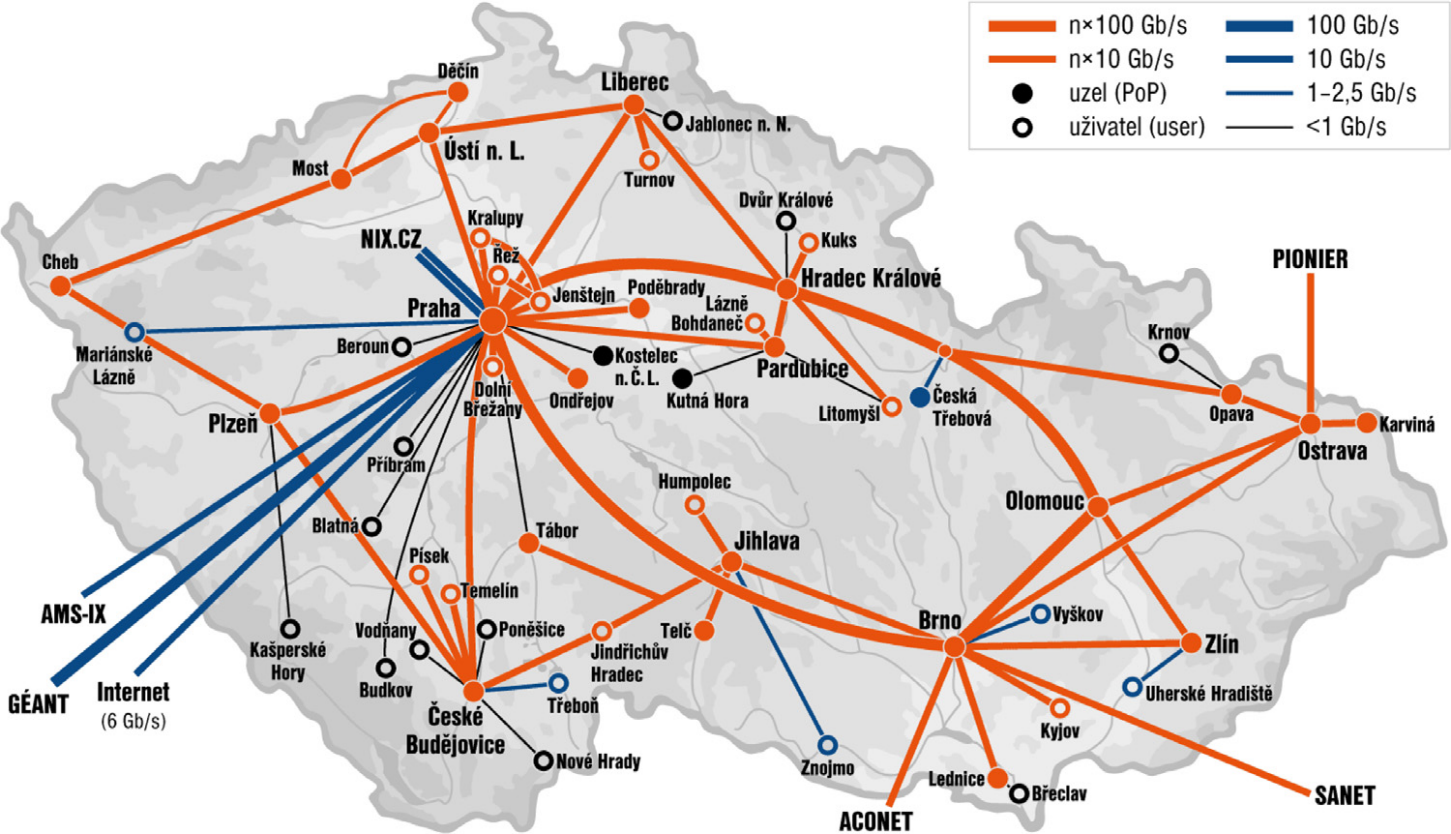
The topology of the CESNET2 network.

**Fig. 5:**
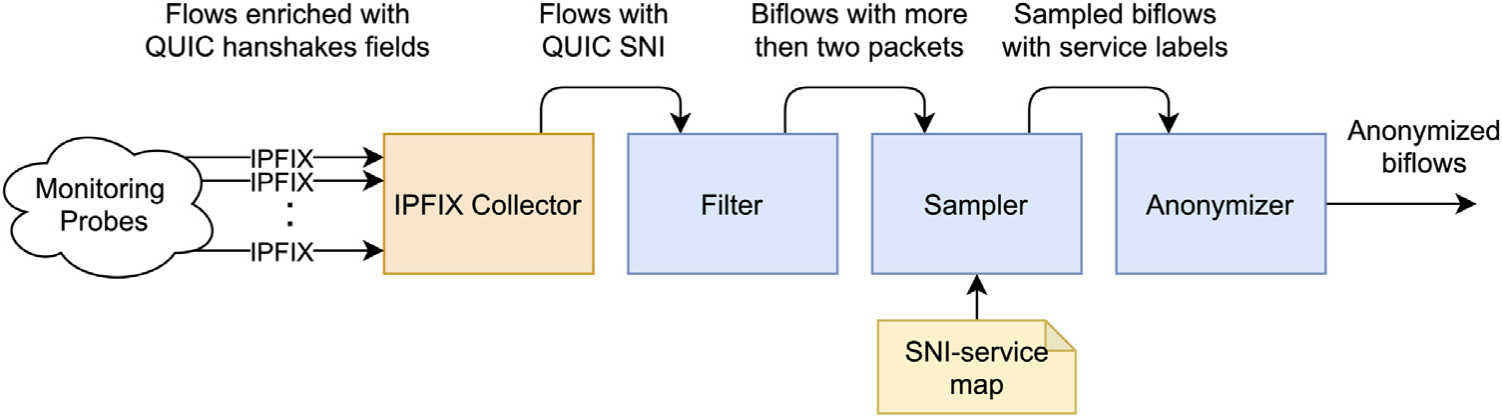
The workflow of the automatic data capture and processing.

The selected services were recognized using Server Name Indication (SNI) domains that were extracted from the QUIC connection handshake. To find the domains associated with a service, we searched its online documentation. In some cases, we found a docpage with “whitelist domains for network and firewall settings”, which contained all the service’s domains. In other cases, we used Netify’s Application Lookup Tool.^5^ For the rest, we analyzed SNI values observed in the network and handpicked the domains. The created SNI–service mapping was used for assigning labels to the captured flow data. Each selected web service represents its own traffic class that can be used in traffic classification tasks. Moreover, we organize services with the same provider into groups, such as Google services or Facebook (Meta) services, to allow service-provider classification tasks.

On top of the selected web services, we also captured background traffic—i.e., traffic not belonging to none of the selected services. The background traffic allows network classifiers to be evaluated in open-world scenarios, where the classifier needs to deal with traffic classes that were not available during training. We decided to split the background traffic into three classes. The Google Background class contains the traffic of not-selected Google services, Facebook Background contains the traffic of not-selected Facebook services, and Default Background contains the traffic of the remaining QUIC web services that are present in the CESNET2 network. This separation allows the evaluation of more granular and detailed open-world scenarios.

### 3.2 Flow enrichment and export

Each monitoring point was installed with ipfixprobe,^6^ which is a high-performance bidirectional flow exporter capable of processing 100 Gbps traffic while exporting extended flow features. We used ipfixprobe’s QUIC plugin, which performs deep packet inspection. When the QUIC plugin detects a QUIC connection handshake, it enriches the flow with the SNI domain, QUIC version, and user agent if available. We also used the PSTATS plugin, which exports metadata statistics (size, direction, and inter-packet time) about the first 30 packets of each flow. The length of 30 is a tradeoff between the performance and link bandwidth limitations and the need for longer sequences for more accurate predictions. The last used plugin was the PHISTS plugin, which exports histograms of packet sizes and inter-packet times of each flow (histograms are not limited to the first 30 packets).

The flow exporting process was set with an active timeout of 5 min and an inactive (idle) timeout of 65 s. Long connections are split when the connection duration is longer than the active timeout, and a flow record is exported even though the actual connection is not terminated yet. If no packet is observed within the inactive timeout period, the connection is considered terminated, and a flow record is exported. Using active and inactive timeouts for splitting connections is standard practice for flow-based network monitoring [12].

Exported flows from each monitoring point were transmitted using the IPFIX protocol to a single flow collector, which performed additional processing.

### 3.3 Flow collection and filtration

Flows were collected using the IPFIXcol2^7^ flow collector, which was executed with a configuration to receive, process, and save flows enriched with QUIC fields. All IPFIX data were converted using IPFIXcol2 and passed into the NEMEA framework,^8^ which allows stream-wise and efficient flow processing.

We performed flow filtration using a NEMEA filtering module.^9^ The filtering module selected QUIC flows that had the destination port 443/UDP, had the QUIC SNI field filled, and had at least one packet in both communication directions to filter out unidirectional flows. Unidirectional flows can be formed in the network due to service scanning, connection errors, or other network phenomena such as asymmetric routing.

Next, flows were assigned a web service (or background) label using the SNI–service mapping described in Section 3.1. Bidirectional QUIC flows with service labels were then passed to the sampling stage.

### 3.4 Sampling

Since our goal is long-term flow capture from a large backbone network, we need to use data sampling to maintain a reasonable size of the dataset. Instead of using one sampling ratio for all traffic, we decided to use a dynamic sampling ratio for each service to soften the class imbalances in the dataset. Each service is sampled at a different ratio, depending on the amount of traffic (i.e., the number of flows) it generates. This dynamic sampling approach ensures that even less-prevalent services are represented in the dataset with a sufficient amount of samples.

Services are sorted based on the amount of their traffic. The top 5% of services are sampled with a 1:15 ratio; the bottom 60% of services are not sampled at all. The rest 35% are sampled with a ratio ranging between 1:2 and 1:9, depending on their prevalence. Moreover, the background traffic classes (see Section 3.1) are all sampled with a 1:15 ratio. The amount of traffic of each service was monitored during the dataset capture, and the sampling ratios were updated every five minutes. The resulting per-service sampling ratios are included in the dataset. It is up to the users of the dataset to decide, depending on their goals, whether to use the dataset’s service distribution with softened imbalances or resample it back to the original CESNET2 distribution.

### 3.5 Anonymization

To protect the privacy of CESNET2 users, we anonymized client IP addresses using the Crypto-PAn (Cryptography-based Prefix-preserving Anonymization) algorithm [13]. Crypto-PAn is an anonymization algorithm for IP addresses that maintains the prefix (subnetwork) structure. We also stripped other fields that could lead to user identification, such as MAC addresses. After the anonymization process, there is no link between dataset flows and actual users, and deanonymization is impossible.

## Ethics Statements

Maintaining the privacy of Internet users is our prime concern; hence, the research and dataset collection were done with extreme carefulness. The indisputable advantages of real traffic generated in a production network come with the cost of potential privacy violations of real users. Therefore, we used automatic data processing with immediate data anonymization. With this, we declare that we did not analyze or process deanonymized data and did not perform any procedures that could lead us to users’ identities.

## CRediT authorship contribution statement

**Jan Luxemburk:** Conceptualization, Data curation, Methodology, Software, Visualization, Validation, Writing – original draft, Writing – review & editing. **Karel Hynek:** Conceptualization, Methodology, Software, Validation, Writing – original draft, Writing – review & editing. **Tomáš Čejka:** Conceptualization, Project administration, Funding acquisition, Supervision. **Andrej Lukačovič:** Software, Validation. **Pavel Šiška:** Software, Validation.

## Declaration of Competing Interest

The authors declare that they have no known competing financial interests or personal relationships that could have appeared to influence the work reported in this paper.

## Data Availability

CESNET-QUIC22: a large one-month QUIC network traffic dataset from backbone lines (Original data) (Zenodo).

## Footnotes

2 https://www.iana.org/assignments/quic/quic.xhtml - user_agent transport parameter.

3 More information in the PHISTS plugin documentation https://github.com/CESNET/ipfixprobe/tree/master#phists.

4 https://www.iana.org/assignments/ipfix/ipfix.xhtml#ipfix-flow-end-reason.

5 https://www.netify.ai/resources/applications.

6 https://github.com/CESNET/ipfixprobe.

7 https://github.com/CESNET/IPFIXcol2.

8 https://nemea.liberouter.org.

9 https://github.com/CESNET/Nemea-Modules/tree/master/unirecfilter.

## Footnotes

1 The closest dataset is the UC Davis QUIC dataset [4], which contains 6.5 K flows and five classes.
